# 320 Diagnostic stewardship of a multiplex pneumonia PCR panel leads to cost savings and more appropriate patient care

**DOI:** 10.1017/ash.2026.10667

**Published:** 2026-06-23

**Authors:** Rachel Ronne, Alexander Kracker, Cassandra Salgado

**Affiliations:** 1 Medical University of South Carolina

## Abstract

**Background** Timely healthcare personnel (HCP) response during occupational exposure investigations is crucial for risk stratification, prophylaxis, and preventing secondary transmission. Non-response delays interventions, creates blind spots, and increases patient and workforce safety risks. Understanding pathogen-specific response patterns can inform targeted strategies to improve engagement and mitigate these risks. Objective To quantify pathogen-specific response and non-response rates among potentially exposed HCP and assess timeliness of engagement during occupational exposure investigations. Methods We conducted a retrospective review of occupational exposure investigations tracked in REDCap between November 2024 and October 2025 at an academic health system. Incidents involving Mycobacterium tuberculosis (TB), Neisseria meningitidis, Bordetella pertussis, and varicella-zoster virus (VZV) were included. HCP response was defined as reviewing exposure details and submitting required attestation online; non-response was failure to complete these steps. Data were extracted October 29, 2025. Descriptive statistics summarized response patterns. Comparisons and calculations of odds ratios (ORs) with 95% confidence intervals (CIs) were performed using chi-square analysis. Results Among 1,442 HCP notified of a potential occupational exposure, overall response was 90.5% (n=1,305) and non-response was 9.5% (n=137). Response within 5 days was 70% (n=1,003). Pathogen-specific response rates varied. HCP with a TB exposure (n=803) had 60% response within 5 days and 90% total response. Pertussis exposures (n=331) had 87% response within 5 days and 97.6% total response. VZV exposures (n=298) had the lowest engagement, with 77% response within 5 days and 83.6% total response. Meningitis exposures (n=10) demonstrated 90% response within 5 days and achieved 100% total response. When comparing response rates by pathogen, pertussis had significantly higher response rates compared to TB (OR 0.22, 95% CI 0.11–0.47, p < 0.001) and VZV (OR 7.95, 95% CI 3.70–17.08, p < 0.001). TB responses were significantly lower than VZV (OR 1.78, 95% CI 1.21–2.61, p = 0.004). Similar trends were observed for 5-day response rates. Conclusion Statistical comparisons underscore disproportionate engagement challenges concentrated in TB and VZV exposures. TB required significant additional outreach to achieve overall response, reflected in the gap between the 5-day response (60%) and final response (90%) values. VZV had the highest non-response rate (16.4%), though clinical impact may be mitigated by workforce immunity. HCP potentially exposed to pertussis and meningitis demonstrated outstanding response, 97.6% and 100% respectively, perhaps due to urgency for post-exposure prophylaxis when indicated. Pathogen-specific engagement strategies are warranted where delayed or absent responses pose safety risks.

